# Application of virtual reality in dental implants: a systematic review

**DOI:** 10.1186/s12903-023-03290-7

**Published:** 2023-08-28

**Authors:** Elham Monaghesh, Ramin Negahdari, Taha Samad-Soltani

**Affiliations:** 1 Department of Health Information Technology, School of Management and Medical Informatics, Tabriz University of Medical Sciences, Tabriz, Iran; 2 Prosthodontics department, Dentistry faculty of tabriz medical university, Tabriz University of Medical Sciences, Tabriz, Iran

**Keywords:** Virtual reality, Dental implants

## Abstract

**Background and objective:**

A treatment approach that is widely used as a permanent and natural replacement for missing or extracted teeth is dental implants .VR is a computer-generated simulation that creates a three-dimensional (3D) image or environment. Advances in VR -based learning allow learners and students to practice and also help professionals plan a wide variety of surgical procedures, including the correct placement of dental implants. Therefore, in this systematic review, our aim was to investigate and evaluate the available virtual reality tools for dental implants and their effectiveness.

**Materials and methods:**

Studies published up to 01/30/2023 which report the applications of using virtual reality technology in dental implants, were reviewed in three databases, including PubMed, Web of Science, and Scopus. All studies with evidence reporting the role of virtual reality technology in the field of dental implants were included in our analyses, written in English and published in peer-reviewed form, are included. Theoretical articles, and letters that did not provide original data, as well as studies that reported incomplete information, were excluded. Two reviewers independently assessed search results, extracted data, and assessed the quality of the included studies, and decisive agreement was reached by discussion and consultation with the third researcher. Narrative synthesis was undertaken to summarize and report the findings.

**Results:**

Out of 1633 initial search results, nine were included in the present study based on the inclusion criteria. The focus of seven studies was on teaching and learning, and two studies have examined the implant planning procedure. The most commonly used hardware and software were head-mounted display and Unity3D, respectively. In almost all studies, the results showed that the use of virtual reality-based systems improves and enhances the skills of users, including dental students and specialists.

**Conclusions:**

Our findings showed that VR is an effective method for teaching and planning the implant process. Although the use of VR technology is limited for various reasons such as cost, it can increase the skills of dental professionals in performing dental implants.

## Introduction

Teeth are sensory organs that play an important role in several different aspects of daily life, including chewing, talking and even smiling [1]. Consequently, they are important organs that are related to quality of life [2]. Unfortunately, these vital organs are not invincible and can be destroyed during a person’s lifetime for a number of reasons. Periodontal disease, caries and dental trauma can all lead to tooth loss at any age [3, 4].

A treatment approach that is widely used as a permanent and natural replacement for missing or extracted teeth is dental implants [5–7]. In general, the long-term survival rate of dental implants is excellent [8]. Also, Oral implantology is an interdisciplinary subject that integrates multidisciplinary knowledge with theoretical knowledge and clinical practice skills [9].

Inaccuracy and improper implant placement can lead to unsuccessful implant restorations or other complications, including implant fracture, loosening, infection, inflammation, bone and soft tissue weakness, and damage to surrounding structures [10, 11]. Also, fracture of the implant body is a very rare event after treatment, which is usually caused by excessive pressure or improper dental prosthesis that transfers pressure from the center to the periphery [12–14]. which can be due to the negative effect of the lack of training on the performance of specialists during the various stages of the implant [11].

In recent years, virtual reality and interactive digital simulation have been used in dental education to train dental students before interacting with real patients. Scientific evidence has provided the use of virtual reality technology in dental education, and some recent publications show that virtual technologies can have positive effects on the results of dental education [15]. VR has been recognized as a valuable tool for dental student education, and its use by dental schools around the world is increasing [16, 17]. Advances in simulation-based learning allow learners and students to practice and also help professionals plan a wide variety of surgical procedures, including the correct placement of dental implants [18]. VR is a computer-generated simulation that creates a three-dimensional (3D) image or environment. VR environments range from full immersion where people can physically interact with virtual objects and people to non-immersive levels [19]. Users wear a head-mounted display that immerses them in an experience where they can interact with the environment and virtual characters in a realistic way. VR can be effective in dental education, allowing a training environment to be provided without direct patient contact [20, 21].

In recent years, several reports have systematically reviewed the results of research investigating the effects of virtual reality-based tools on various aspects of dentistry. But there is no study that reviews the use of virtual reality in dental implants. Therefore, in this systematic review, our aim was to investigate and evaluate the available virtual reality tools for dental implants and their effectiveness. In other words, the effectiveness of VR-based interventions for dental implants was investigated, and the key features of these interventions were identified in relation to their effectiveness.

## Materials and methods

This review was reported in accordance with the Preferred Reporting Items for Systematic Reviews and Meta-Analyses (PRISMA) guidelines. The study was designed as a systematic review.

This systematic review was performed using the Population, Intervention, Comparison, Outcomes, and Study (PICOS) framework to recognize proper sources and to search for related evidence to form a focused question and facilitate literature search. In this framework, the population includes specialists, students and people working in the field of dentistry. This intervention required the use of virtual reality tools with comparison groups that included the use of non-VR-based tools, conditions before using VR. In addition, the outcome included the effect of using virtual reality for dental implants.

### Literature search

In order to identify relevant and published studies, four important medical and health databases including PubMed, Embase, Web of Science, and Scopus were searched on 10/18/2022. To update the results, a second search was conducted on 01/30/2023. The search was performed on titles and abstracts. Also, the search for studies was done without time limit. In addition, in order to identify additional studies not identified during the electronic database search, the references of all included studies were manually searched. A combination of keywords and medical subject headings (MeSH) was used for searching, which includes: dental, oral, implant, VR, virtual reality, virtual realities, Virtual Environment, Simulation. Boolean operators (AND, OR and NOT) were also used to combine terms. At this point, a librarian was consulted to confirm that the search strategy was satisfactory. The search in each database was then adapted accordingly. For example, the search strategy in the PubMed database was applied as follows:

(dental[title/Abstract] OR oral[title/Abstract]) AND (implant[title/Abstract]) AND (VR[title/Abstract] OR “virtual reality“[title/Abstract] OR “Virtual Environment“[title/Abstract] OR “virtual realities“[title/Abstract] OR Simulation[title/Abstract])

Then, to remove duplicates, the results obtained from different sources were checked and entered into the Endnote resource management software.

### Inclusion and exclusion criteria

All studies with evidence reporting the role of virtual reality technology in the field of dental implants were included in our analyses. Also, studies that were published up to 01/30/2023, written in English and published in peer-reviewed form, are included. In fact, they were included if they clearly described the type of virtual reality function in dental implant education, diagnosis, management, and treatment., theoretical articles, and letters that did not provide original data, as well as studies that reported incomplete information, were excluded.

### Study selection

First, duplicate references were removed, then screening was done in two steps. In the first step, the titles and abstracts of the articles were reviewed to identify relevant studies based on the eligibility criteria. In the second step, after the initial screening, two researchers separately reviewed the full text of the articles that were identified based on the relevant criteria. The agreement regarding the disagreement of the scholars in the selection of articles was reached by discussion and consultation with the third researcher.

### Quality assessment

The Critical Appraisal Skills Program (CASP) checklist tools were developed to teach people how to critically evaluate different types of evidence (NCCf, 2011). To assess the quality of included studies, a standard CASP randomized controlled trial checklist was provided [46]. The included studies were divided into three categories, poor, medium and good, based on the quality score. All articles, regardless of methodological quality, were subjected to data extraction and synthesis due to the technical and developmental nature of the original articles. Therefore, we did not exclude reviews that were of good technical quality but did not meet the requirements of the checklists [47].

### Data extraction

Selected studies were thoroughly reviewed and data were extracted from all articles that met the eligibility and inclusion criteria. The data was extracted by a researcher using the designed data extraction sheet and verified by the second researcher. Selected data were publication data (i.e. author, year and country), study objective, study design, sample size, participant data (i.e., age, gender), Target population (patients or students or new graduates or doctors), technology used, scenario, data on methodology, assessment (i.e., time, test and scores), and results.

### Evidence synthesis

Data of the included studies was qualitatively described and presented. The authors to reach consensus on the findings, met frequently to discuss.

## Results

### Study selection

**Fig. 1:**
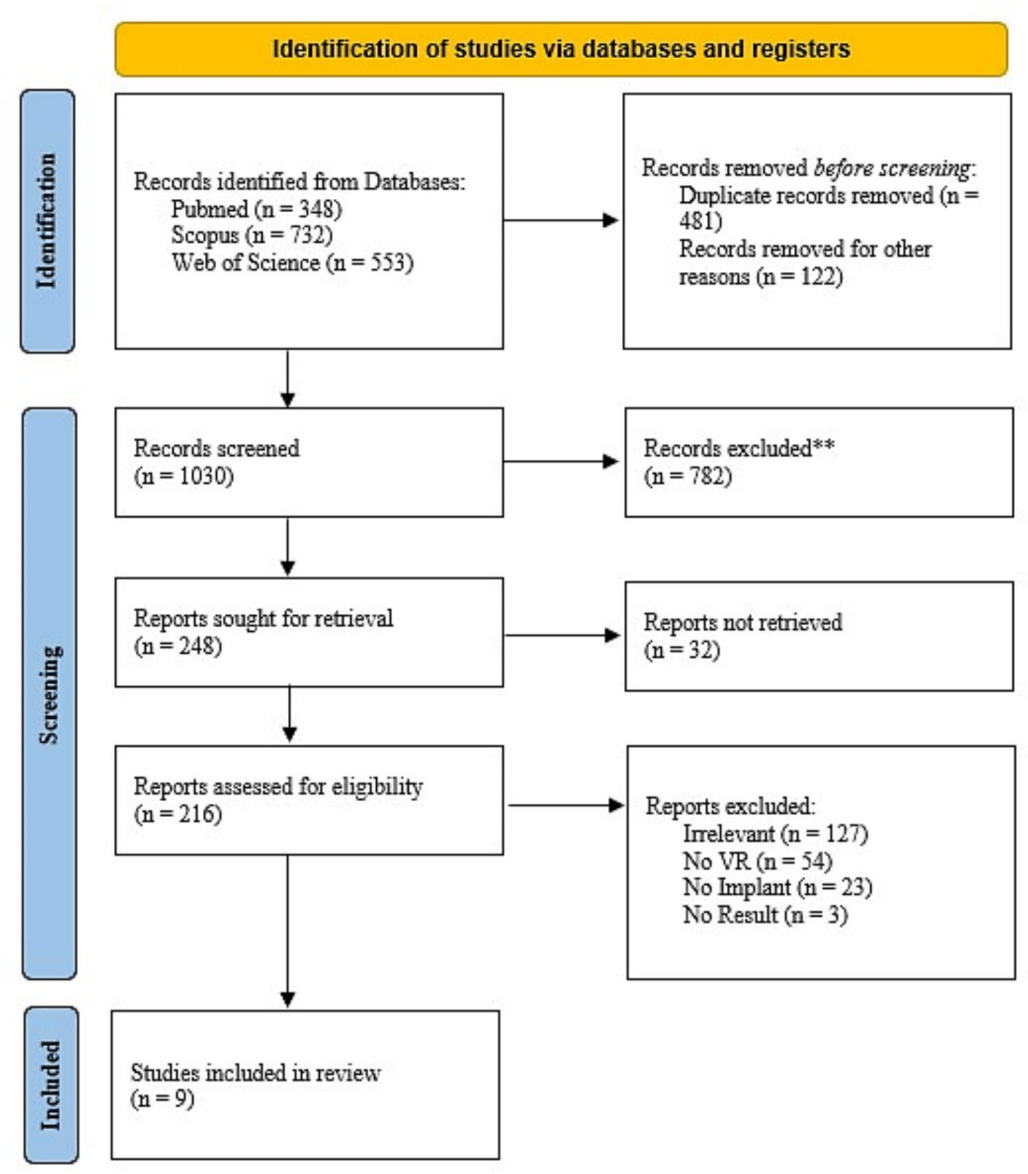
PRISMA flow diagram illustrating study selection

In order to develop a repeatable approach, a systematic review method was used. This study review was conducted pursuant to the Preferred Reporting Items for Systematic Reviews and Meta-Analyses (PRISMA) guidelines. The flowchart of the study selection process is shown in Fig. 1, which shows the number of studies obtained from each database, the number of screened studies, excluded studies, and finally the included studies. At first, 1633 studies were identified and after removing duplicates and screening based on their titles and abstracts, the remaining articles were selected for full text review. In the next step, conference abstracts, review studies, topics that were irrelevant and studies that did not report results were excluded. Also, one article for which the full text was not available and no response from the authors was received was excluded (Trenanas et al., 2009). Finally, nine studies remained for review in this study.

### Characteristics of studies

The full characteristics of the included studies are shown in Table 1. The included studies published in various international journals between 2006 and 2022. Nine included studies were carried out in seven countries: China (n = 2), Finland (n = 2), USA (n = 1), Japan (n = 1), Portugal (n = 1), Germany (n = 1) and Israel (n = 1). Based on the type of study, all studies were original research. The general aim was to identify the areas of using virtual reality in dental implants and to evaluate the effectiveness of using virtual environments in the skills of people engaged in dental implants. Among the included studies, the focus of seven studies was on teaching and learning, and two studies have examined the implant planning procedure. Participants in the included studies dental professionals, young physicians and graduate students, dentomaxillofacial radiologists, novice surgeon, and residents.

### Quality of the included studies

Our systematic review consisted of nine studies that were evaluated using CASP tools. The quality of the evaluated studies were generally high level. six (66%) studies were of good quality and three (33%) studies were of medium quality. Also, no studies were excluded on the basis of the level of quality appraisal.

**Table 1:** Summary characteristic of included studies in systematic review

No	Author, year, Country	Aim of study	Domain	Sample size		Participants
				experiment	control	
1	Yong Zhou 2021 Chinan [22]	To evaluate the application of virtual reality technology in a dental implant training system.	Training	15	15	Young physicians and graduate students
2	Naoki Kusumoto 2006 Japan [23]	To investigate the accuracy of the bone-supported surgical pattern performed using a virtual reality force feedback haptic device to enable a person to feel the tactile sensation of drilling the jawbone in implant operations.	Training	-	-	Dental professionals.
3	Hanna-Riikka Rantamaa 2022 Finland [24]	Evaluation of voice commands for mode change in virtual reality implant planning procedure	Implant planning procedure	3	3	Dentomaxillofacial radiologists
4	Ezequiel Roberto Zorzal 2021 Portugal [25]	To verify if “IMMPLANT” can positively assist teaching and learning experiences for planning implant placement procedures	Training and education	16		Dental professionals
5	Cortino Sukotjo 2021 USA [26]	Describe the development of virtual reality for implant surgery, and to investigate student perception of the use of VR in teaching dental students dental implant placement.	Teaching and education	7		Dental students enrolled in the Advanced Predoctoral Implant Program (APIP)
6	Xiaojun Chen 2018 China [27]	Present a haptic simulator for trainees to study and rehearse the drilling performance of dental implant surgery.	Study and rehearse	30		Novice surgeon
7	Pohlenz, P. 2010 Germany [28]	Evaluation of dental students’ perceptions of simulator teaching technique in dentistry, virtual apicectomy in “Voxel-Man” simulator.	Training	53		Dental students without practical experience of performing real surgery, who had previously used the standard established phantom environment of plastic and rubber.
8	Nardy Casap 2011 Israel [29]	Assess the students’ performance in dental implantation assignments by comparing freehand protocols with virtual reality navigation.	Teach and train	20	20	Final-year undergraduate dentistry students without experience in dental implantation surgery
9	Hanna-Riikka Rantamaa 2022 Finland [30]	Evaluation of virtual handles for dental implant manipulation in virtual reality implant planning procedure	Implant planning procedure	8		Four dentomaxillofacial radiologists, two seniors, with experience in dentistry for 36 and 23 years, and two residents, with experience in dentistry for 8 years.

### Virtual reality

The VR technology creates a virtual environment using tools that create a situation, image, and sound meant to enhance the sense of presence. A summary of the technology used in the reviewed studies is described in Table 2. The most common hardware used included head-mounted displays (HMDs), haptic devices, touch controllers, and smartphones. In one research, used an immersive head-mounted display, a small hand tracking device and a smartphone that are all connected to a laptop.

Unity3D was the most frequently used software. The designed scenarios included the environment of a operating room or dental office, and the skull and upper or lower jaw were designed to simulate the inside of the patient’s mouth. For more realistic simulation, the dental tools and equipment needed to perform the implant process, including a drill for drilling, have also been designed so that users can perform the drilling and implant processes through interaction with the designed virtual environment. More details are shown in Table 2.

### Outcomes

In almost all studies, the results showed that the use of virtual reality-based systems improves and enhances the skills of users, including dental students and specialists. Considering that most of the studies were aimed at teaching and learning, the results of the studies showed that virtual reality is potentially usable for oral implant surgery and is a promising tool to help students learn and 3D dental visualization for teaching implant placement. This software allows students to fully immerse themselves in virtual dental surgery. It can also provide learning and teaching opportunities in the curriculum and preclinical exercises. The evaluations showed that students recommend virtual simulation as an additional method in dental education and are satisfied with its use. The results of two studies that were conducted with the aim of implant planning procedure showed that virtual reality was accepted by experts and they were able to plan several implants satisfactorily. More details are shown in Table 2.

**Table 2:** Intervention characteristic of included studies in systematic review

Author, year, Country	Technology used		Scenario	Methodology		Evaluation		Results
	Hardware	Software		Duration	Place	Evaluation time	Evaluated items	
Yong Zhou 2021 Chinan [22]	HTC vive helmet and its matching handle, Straumann implant surgical toolbox	3Ds Max, Unity 3D	The digital model includes the basic configuration of the operating room and commonly used surgical instruments. When virtual surgery is in progress, the operator needs to press the handle button to achieve the picking action and feel the components’ existence and movement.	1-month	The Stomatological Hospital of Fujian Medical University.	After training	Fidelity and user-friendliness of the simulator, and other questions were designed according to the detailed surgical procedure.	A set of virtual surgery system for dental implant training, which can be used for teaching and training, with good operability and predictability, to achieve a breakthrough in dental implant surgery training.
Naoki Kusumoto 2006 Japan [23]	Haptic device PHANToM	FreeForm	The 3-D bone STL data were input to software, and operation was conducted with the virtual reality force feedback haptic device shown. VRDTS software for virtual tooth preparation was applied, and virtual experience of bone drilling was realized.	-	**-**	-	Cross-sections of the guide holes in the surgical template were imaged, and misalignment between the guide holes of the surgical template and the drilled holes on the jawbone was measured.	The present system is potentially applicable to oral implant surgery.
Hanna-Riikka Rantamaa 2022 [24]	Oculus Quest 2, Touch controllers	Unity 3D	The task was to do the planning of two implants for a dental bridge. This was more demanding than finding a location for a single implant as two implants need to be aligned and suitably located. The implants were moved to their planned positions by picking them up using a controller and doing the necessary translations and rotations. For the experiment, we prepared a 3D skull model.	-	**-**	Twice	Implant placement times, the final implant positions, the number of implant pickups and releases, and the number of times that the participants were using the voice commands.	The tool was accepted by the experts and they were able to do multiple-implant planning satisfactorily. The voice commands were useful, natural, and accurate for mode change, and they could be expanded to other tasks.
Ezequiel Roberto Zorzal 2021 [25]	An immersive head-mounted display, a small hand tracking device and a smartphone that are all connected to a laptop.	Unity 3D	Participants were asked to place a virtual implant at a specific bone loss area location within a subject-specific 3D model of a lower jaw. In particular, participants were requested to perform the implant placement tasks by, firstly, placing the implant as close as possible to the predetermined location and, secondly, by adjusting the position and inclination with finer input through thumb gestures.	20–30 min	**-**	-	Usefulness, ystem Usability Scale, satisfaction, and user acceptance of IMMPLANT’s interactive and immersion features. The capability of " IMMPLANT " in increasing 3D visualization and manipulation of anatomical content in implantology education.	“IMMPLANT” is a promising virtual reality tool to assist student learning and 3D dental visualization for implant placement education.
Cortino Sukotjo [26]	Oculus Quest controller, Front Facing Cameras, the latest untethered VR HMD or headset manufactured by Facebook, Oculus Quest.	Unity 3D	Once the users immerse into the virtual surgical room, they will be able to walk around the scene and explore it. The operatory room is the main room where the user can interact with the patient and practice the implant surgical procedure.	10–15 min	**-**	Before-After training	The applicability and usability of the VR program in the curriculum	This application allows the students to be immersed fully with virtual dental operatory. This application can offer learning and teaching opportunities in the preclinical curriculum and exercises, especially during these challenging times (covid-19).
Xiaojun Chen 2018 China [27]	Omega.6	Visual Studio 2010	A patient-specific drilling simulator based on virtual reality for dental implant surgery is presented. The simulation of stepwise drilling was conducted, and three patient-specific models reconstructed by Computed Tomography data were employed to help the novices to find the suitable drilling parameter.		Shanghai Jiao Tong University School of Medicine	After experiencing the “DISS”	Usability, visual and haptic authenticity. The usability refers to convenience of learning, friendliness of human–machine interface, and convenience of operation. The visual authenticity includes verisimilitude of the anatomical and surgical instrument models and the immersion of virtual environment. The haptic authenticity involves the stability and real-time performance of the force feedback. Also conducted an evaluation study regarding the time consumption.	The obtained results showed that the haptic-based “DISS” could simulate various dental implant surgeries with different driller diameter and drill speed which takes patient-specific models as input. The evaluation of the DISS proves its good performance and it could provide an effective method to improve the skills and experiences of trainees.
Pohlenz, P. 2010 Germany [28]	Voxel-Man simulator, shutter glasses, foot pedal, Phantom Desktop force feedback device, mirror	-	To pilot-test this virtual reality training method for oral surgery. Using a Phantom Desktop force feedback device the user can control a simulated drill, at the same time feeling the forces resulting from the drilling process. Operators see the scene displayed in stereoscopic mode through a mirror. He can also navigate the drill via the three orthogonal CT slices, which change their positions with the drill. A list of mistakenly touched objects is displayed.	-	**-**	After students performed virtual apicectomie	The Voxel-Man simulator was evaluated using a ranking scale regarding the value of virtual reality as an additional educational modality resolution, simulated force feedback, spatial 3D perception, resolution and the integration of further pathologic conditions. students provided their impressions after virtual simulation of apicectomies in the Voxel-Man simulator.	The evaluation of the questionnaire showed that students recommended the virtual simulation as an additional modality in dental education. The operating field can be magnified up to 20-fold, allowing the simulation and performance of surgical procedures under microsurgical conditions.
Nardy Casap DMD 2011 Israel [29]	Camera, patient tracker and the handpiece tracker	The Denex Image Guided Implantology (IGI) software using a version (3.6.5.1)	Final-year dentistry students without previous experience in dental implantation surgery were given an implantation assignment comprising 3 tasks. Marking, drilling, and widening of implant holes were executed by a freehand protocol on the 2 mandibular sides by 1 group and by virtual reality navigation on 1 side and contralaterally with the freehand protocol by the other group.	1 training session	**-**	After completing the assignments	Subjective and objective assessments of the students’ performance, the degree of improved performance, performance on the 2 bilateral assignments and the degree of learning dental implantation from theexercise.	The execution of all assignments was significantly faster in the freehand group than in the navigation group
Hanna-Riikka Rantamaa 2022 Finland [30]	Oculus Quest 2 HMD and controllers	Unity 3D, Planmeca	The skull models were presented in the same order. In the first skull model, the implant was positioned to the left side (patient view) of mandible bone. In the second model, the implant was positioned to right side of mandible and in the last model to the front of the maxilla bone.Two participants did the implant planning first with Handles condition and then Without handles. The two others did it the other way around.	Practice and the questionnaires, took around 45 min	**-**	During the process	To evaluate the usability of the interaction methods, three dimensions were investigated: 1)Efficiency: Task completion time 2)Effectiveness: Marking consistency 3)Satisfaction: Subjective evaluations Measured the task completion times from the moment that the implant was picked up to the moment that the participant released the implant for the last time. For the marking consistency, measured the positions of each implant and compared the positions in the analysis phase. Also the number of implant pick ups and releases was counted .	All Four dentomaxillofacial radiologists ranked direct interaction, planning the implant placement without handles, to be better than the indirect condition where the implant model had handles. The radiologists valued the three-dimensional environment for three-dimensional object manipulation even if usability issues of the handles affected the feel of use and the evaluation results. Direct interaction was seen as easy, accurate, and natural.

## Discussion

In dental implantology, accurate placement of dental implants is necessary to meet the required functional and aesthetic needs [31].This manuscript reviews the available VR technologies and examines its impact on all aspects of dental implants. According to the reviewed studies, the use of VR in the field of dental implants has different goals, including simulating dental education, student performance in dental implant tasks, studying and practicing dental implant surgical drilling, and virtual reality implant planning. In fact, the studies we reviewed generally investigated the effect of VR-based systems on improving the performance of dental students and professionals.

The use of VR in dental education has evolved significantly and there is increasing scientific evidence describing different virtual settings in different dental education modules. However, the true significance of VR simulation on dental education outcomes is not fully understood. Previously, VR was usually considered a luxury or optional tool, but with the COVID-19 pandemic, there was a need for dental students to continue their curriculum without any hindrance in physical presence. VR may therefore provide an opportunity for dental students to develop and maintain theoretical and clinical dental expertise remotely [15]. In this study, the review of studies showed that most of the studies were conducted with the aim of teaching and learning.

Virtual reality provides the possibility of simulating a virtual environment to evaluate different anatomical areas of the body for diagnosis, planning and surgical training. Also, VR helps to plan implant procedures and surgical training [31]. The importance of virtual reality (VR) is recognized by dental professionals and has revolutionized dental education in the modern world of dentistry. With this new technology, students can interact and learn therapy skills with digital on-screen simulations before transferring them to real-life situations. It is useful for gaining confidence in performing skills, reviewing exercises over and over again without wasting material, and for assessing students controlled by the teacher or instructor. This technology holds promise for enhancing dental education by creating a safe learning environment for the teacher and learner or participant [32].

There have been many advances in simulation-based training that allow learners and professionals to practice and create opportunities to plan a wide range of surgical procedures, including the correct placement of dental implants [18]. Virtual implant planning has the potential to treat complex cases in immunocompromised patients with minimal surgical procedures, so this method has revolutionized implant dentistry [33]. Based on the results of this study, experts were able to satisfactorily plan multiple implants using virtual reality tools, as a result, virtual reality was accepted by them [9].

The literature points to the importance of virtual reality simulation for the standardization of dental education and the need for further studies to optimize the value, assessment method, student feedback and integration mechanism in the dental curriculum. Several methods have been proposed to motivate students towards self-learning, thereby reducing teachers’ time and improving their teaching skills [32].

Based on the results of several previous studies, it has been found that VR significantly increases the acquisition of dental manual skills even in short training periods and, to a lesser extent, the retention of theoretical knowledge [15, 34–36]. According to the results of this study, the implant is a promising virtual reality tool to help students learn and visualize 3D dentistry for teaching implant placement [11]. This software allows students to fully immerse themselves in a virtual dental surgery environment. This program can provide learning and teaching opportunities in pre-clinical curriculum and practice, especially during the challenging period of the covid-19 pandemic [37]. And it can be an effective way to improve the skills and experience of trainees [38]. Also, the evaluation of the questionnaire showed that students recommend virtual simulation as an additional method in dental education [28, 39].

Also, based on the results of a study, the virtual surgery system for dental implant training can be used for teaching and learning, with good performance and prediction, to achieve progress in dental implant surgery training [22]. Also, users were satisfied with using the system and reported that direct interaction with the virtual environment was easy, accurate and real [40]. Therefore, based on studies, it can be concluded that virtual reality is an educational tool for students and even professionals that can be used to improve their skills in dental implant skills.

Also, based on a study, a patient-specific tactile drilling simulator based on virtual reality was performed for dental implant surgery [27]. The obtained results showed that the designed touch-based system can simulate different dental implant surgeries with different drill diameter and drill speed, which also takes patient-specific models as input. The evaluation of this system has proven its good performance and can be an effective way to improve the skills and experience of trainees. Therefore, it can be concluded that the use of VR can provide the possibility for students to study, rehearse and repeat several times, while this is not possible on a real patient.

Considering that two studies were conducted with the aim of planning the implant process [24, 40], the results of these studies showed that this tool was accepted by the experts and they were able to plan several implants satisfactorily. Also, in this study, voice commands were used to perform functions, and it was found that voice commands were useful, natural and accurate for changing the state, and they can be extended to other tasks [24]. In another study, radiologists evaluated the 3D environment for manipulating 3D objects, which showed that the usability of the handles affects the sense of use and evaluation results [40].

VR applied in dental education represents a wide range of applied devices and technologies, from VR simulation with or without immersive environment, tactile simulators with or without force feedback, real-time digital mapping and assessment, various mobile virtual platforms, video games and Other forms of virtual packages. The variation in individual sub-characteristics reflects the fact that there are no recognized training standards for dental simulators or related exercises [16]. Most of the reviewed studies use immersive environments that allow for more settings and a sense of real presence and interaction. VR technology provides a variety of features to increase the sense of immersion in people. In the reviewed studies, different hardware and software have been used to create a virtual environment. HMD and 3DOF tracker were used to move in laboratory environments. Various software are also used for virtual reality design, of which Unity3D is used more. It is not possible to compare these methods because they all achieved favorable results or measured different outcome measures.

Also, one study used tactile simulation [23]. It was found that tactile technologies were used in few studies. While the use of tactile technologies, especially in tasks that require drilling and tooth preparation, provides another dimension to VR by creating a sense of touch and force feedback in the different layered structure of the tooth and bone [16]. It can also help students improve preparation accuracy, and reinforce a conservative preparation approach [41, 42]. Therefore, due to the unique characteristics of dental procedures, force feedback sensing should be improved and included as an integral feature in any dental training simulator to increase understanding of tooth structure and different bone layers [43]. In fact, training with force feedback leads to creating a sense of realism in the user and allows the learner to get the feeling of a real environment in a virtual learning environment.

Therefore, VR showed wide applications in dental implant training and has a significant positive effect on manual skills and theoretical knowledge acquisition. Also, VR reduces the anxiety associated with real patient management during the implementation of a treatment plan, because it exposes students to an interactive learning experience and increases the possibility of self-evaluation, and ultimately leads to increased self-confidence when dealing with real patients. Also, given that simulators are flexible in terms of time, students can repeat procedures to achieve an acceptable skill level without trespassing on real patients and eliminating the need for long-term direct contact [34, 35, 44].

Based on the results of this review, it is recommended that low-cost VR hardware and software become readily available to create safe and cost-effective interactive training, allowing learners and trainees to interact in real-time via their personal computers or mobile phones. Apart from the virtual content, it is recommended to clarify the training content and the extent to which conventional workflows should be taught.

To evaluate the virtual reality in the identified studies, three things have been examined in most of the studies, which include user-friendliness of the system, usability and user satisfaction [9, 37, 39, 45]. In a study to evaluate the usability of interaction methods according to the ISO definition, three dimensions were examined, including efficiency (task completion time), effectiveness (marking consistency) and satisfaction (subjective evaluations). In fact, the time to complete the task was measured from the moment the implant was removed to the moment the participant released the implant for the last time. Also, the number of implants removed and released was counted [40]. It also refers to ease of learning and ease of performance [37, 39, 45]. Also, to evaluate the functional skills of the dental implant, the duration of implant placement, the final positions of the implant, the number of removal and release of the implant, and the number of times the participants used voice commands [45]. Among other things evaluated, the visual and tactile originality of the virtual environment. was designed Visual authenticity includes accuracy of anatomical and surgical instrument models and immersion in the virtual environment. Tactile authenticity includes stability and real-time performance of force feedback [38]. An additional training method clarity study assessed simulated force feedback, 3D spatial perception, clarity and integration of further pathological conditions. Students presented their impressions after the virtual simulation of apicectomy in the “Voxel-Man” simulator [39]. Therefore, subjective and objective evaluation of students’ performance regarding the use of virtual reality has been done with a focus on dental implants. Also, the degree of performance improvement after using the system, and compared with the traditional method. Questionnaires and surveys were used to evaluate these items [29, 37, 39, 40, 45].

Thus, VR provides an opportunity for clinicians to perform safe dental implant placement in a virtually realistic environment with constant feedback. Dental simulators mimic anatomical structures along with creating all tactile sensations. The evolution of technology in the modern world has made virtual reality simulation-based training an integral part of learning for dental students. Additionally, in the era of COVID-19 when social distancing is a necessity, dental education should be conducted in a way that avoids unnecessary interactions, therefore, institutions should emphasize the importance of simulators. Haptic machines give students the opportunity to practice a wide range of clinical skills in a safe environment and increase their confidence before transferring to patients, which also benefits patients.

The purpose of including new technologies such as virtual reality and touch in dental education is to guide and help the development of skills of students and professionals. Therefore, they can be trained with continuous training that will improve their skills in various procedures such as tooth preparation, cavity preparation, prosthesis fabrication and finally implant placement by increasing the tactile sense ability with pre-action feedback mechanism.

Limitations.

The considerable thing about the large number of studies found after the initial search is that we have used the keyword “simulation” in the search strategy. The reason for using this word was to avoid losing relevant studies, but it is clear that this word is used a lot in dentistry for a variety of purposes, which is not related to virtual reality.

A common limitation of all systematic reviews is bias in the selection of articles. Therefore, the search strategy was planned to include all studies in this field. Additionally, only articles published in English were included. We were unable to access the full text of a study as previously described. The number of studies is small, although they show promising effects of using virtual reality in improving the performance of professionals and students. Increasing studies over time allow for comparisons between techniques.

## Conclusions

This review identifies the required features of virtual reality-based systems when used for dental implants. In addition, we presented the evaluations conducted by the studies to determine the effectiveness of the interventions. This study shows that the use of VR in dentistry improves the function and processes of implants. Although the use of virtual reality technology is limited for several reasons, including cost, this method is more attractive than conventional interventions. VR technology improves the performance of dental students and professionals, improves implant process planning. In addition, it improves the quality of education, the opportunity to repeat educational sessions and reduce the risk of student-patient interaction. However, with the advent of cost-effective, user-friendly virtual reality systems with haptic features, more research on VR can be done based on initial studies to develop new applications for VR in implants.

## Data Availability

The datasets generated and/or analysed during the current study are not publicly available due to the non-public nature of the data but are available from the corresponding author on reasonable request.
